# Pharmacokinetics, pharmacodynamics and efficacy of pemigatinib (a selective inhibitor of fibroblast growth factor receptor 1–3) monotherapy in Chinese patients with advanced solid tumors: a phase i clinical trial

**DOI:** 10.1007/s10637-023-01396-x

**Published:** 2023-10-27

**Authors:** Ting Deng, Le Zhang, Yehui Shi, Guiying Bai, Yueyin Pan, Aizong Shen, Xinghua Han, Zhaoyi Yang, Mingxia Chen, Hui Zhou, Yang Luo, Shirui Zheng, Yi Ba

**Affiliations:** 1 Department of GI Medical Oncology, Tianjin Medical University Cancer Institute and Hospital, National Clinical Research Center for Cancer, and Tianjin’s Clinical Research Center for Cancer, and Tianjin’s Key Laboratory of Cancer Prevention and Therapy, Tianjin, China; 2 Phase I Clinical Trial Ward, Tianjin Medical University Cancer Institute and Hospital, National Clinical Research Center for Cancer, & Tianjin’s Clinical Research Center for Cancer, & Tianjin’s Key Laboratory of Cancer Prevention and Therapy, Tianjin, China; 3 Oncology Department, Anhui Provincial Hospital, Hefei, China; 4 Pharmacy Department, Anhui Provincial Hospital, Hefei, China; 5 Department of Biostatistics and Information, Innovent Biologics, Inc, Suzhou, China; 6 Department of Medical Science and Oncology, Innovent Biologics, Inc, Suzhou, China; 7 Department of Clinical Pharmacology, Innovent Biologics, Inc, Suzhou, China; 8 Department of Cancer Center, Peking Union Medical College Hospital, Chinese Academy of Medical Sciences, Beijing, China

**Keywords:** Pemigatinib, Advanced solid tumors, FGFR alterations, Chinese patients, Pharmacokinetics, Pharmacodynamics

## Abstract

Pemigatinib is a selective fibroblast growth factor receptor (FGFR)1–3 inhibitor and has demonstrated acceptable tolerability and clinical activity in advanced solid tumors in Western population. This phase I trial evaluated pharmacokinetics/pharmacodynamics (PK/PD) characteristics, preliminary safety and efficacy of pemigatinib in Chinese patients with advanced, solid tumors. Patients with unresectable advanced or metastatic solid tumors bearing FGF/FGFR1-3 alterations received oral pemigatinib at 13.5 mg once daily (QD) on a 2-weeks-on/1-week-off schedule. The primary endpoint was PK/PD characteristics; secondary endpoints were safety and efficacy. Twelve patients were enrolled (median age: 61 years, 58.3% males). PK data demonstrated pemigatinib (13.5 mg QD) was rapidly absorbed with a geometric mean elimination half-life of 11.3 h. The geometric mean values of maximum serum concentration and area under the plasma concentration–time curve from 0 to 24 h at steady state were 215.1 nmol/L and 2636.9 h·nmol/L, respectively. The mean clearance adjusted by bioavailability at steady state was low (11.8 L/h), and the apparent oral volume of distribution was moderate (170.5 L). The PD marker, serum phosphate level, increased on days 8 and 15 of cycle 1 (mean: 2.25 mg/dL, CV% [percent coefficient of variation]: 31.3%) and decreased to baseline post 1 week off. Three (25.0%) patients experienced grade ≥ 3 treatment-emergent adverse events. Partial response was confirmed in one patient with FGFR1-mutant esophageal carcinoma and one with FGFR2-mutant cholagiocarcinoma. Pemigatinib had similar PK/PD characteristics to Western population and demonstrated an acceptable safety profile and potential anti-cancer benefit in Chinese patients with FGF/FGFR1-3 altered, advanced, solid tumor. *(ClinicalTrials.gov: NCT04258527 [prospectively registered February 6, 2020]).*

**Supplementary Information:**

The online version contains supplementary material available at 10.1007/s10637-023-01396-x.

## Introduction

The fibroblast growth factor (FGF) signaling pathway is associated with oncogenesis of human malignancy [1]. A variety of positive genomic alterations in FGF/FGF receptor (FGFR) have been found in multiple tumor types including gastric cancer (GC) [2], non-small cell lung cancer (NSCLC) [3], urothelial carcinomas (UC) [4], and colorectal cancer (CRC) [5] among others. Although the multi-target tyrosine kinase inhibitors have demonstrated clinical activity in some cancer types, they are limited by off-target toxicity.

In recent years, a great deal of effort has been devoted to develop precise anti-cancer therapeutics by selectively targeting FGFRs: such as FGFR1-4 inhibitors of futibatinib (TAS-120) [6], derazantinib (ARQ 087) [7], LY2874455 [8], and KIN-3248 [9], and FGFR1-3 inhibitors of AZD4575 [10] and Debio 1347 [11] under clinical development. As so far several FGFR inhibitors have been approved by the regulatory agencies. Specifically, erdafitinib, a pan-FGFR inhibitor, was approved by US Food and Drug Administration (FDA) in 2019 in treating adult patients with locally advanced or metastatic UC harboring FGFR3 or FGFR2 genetic alteration based on an overall objective response rate (ORR) of 40% in 99 patients with UC in an open-label phase II study [12]. Infigratinib is a selective FGFR1-3 inhibitor; it showed an ORR of 23.1% in 108 patients with previously-treated, unresectable locally advanced or metastatic, FGFR2 fusion/rearrangement-positive cholangiocarcinoma (CCA) in a single-arm, phase II study [13, 14] and received US FDA approval for this indication in 2021. Pemigatinib is a potent and selective oral inhibitor of FGFR1-3, which has demonstrated encouraging clinical benefit in patients with certain malignancies. Pemigatinib was first approved by US FDA in FGFR2 fusion/rearrangement-positive CCA in 2020 based on an single-arm phase II FIGHT-202 study with a centrally confirmed ORR of 35.5% in 107 patients [15, 16]. Pemigatinib also showed durable and high rates of complete responses and complete cytogenetic responses in patients with relapsed or refractory myeloid/lymphoid neoplasms (RRMLN) with FGFR1 rearrangement in the phase II FIGHT-203 study, based on which it received FDA approval for FGFR1-rearranged RRMLN in 2022 [17].

There is still a large unmet medical need for precision therapy in cancer patients in China, however, none of the selective-FGFR inhibitors were approved in the country when this study was initiated. Although pemigatinib has been deeply investigated in Western population and demonstrated clinical benefit in CCA and RRMLN and antitumor activity signals in several types of solid tumor including pancreatic cancer, gallbladder cancer, UC, etc. with acceptable safety [15, 17, 18], its PK/PD profile, safety, and antitumor activity is barely understood in Chinese patient population.

This phase I, open-label study was conducted to investigate pemigatinib in Chinese patients with FGF/FGFR1-3 altered, advanced, metastatic or recurrent, solid tumors. The primary objective was to assess the pharmacokinetics/pharmacodynamics (PK/PD) of pemigatinib; secondary objectives included safety and efficacy.

## Methods

### Ethical approval

The trial protocol was approved by the ethics committees of two participating hospitals. The study was conducted in accordance with the protocol, Good Clinical Practice Guidelines, Declaration of Helsinki, and local applicable regulatory requirements. Written informed consents were obtained before enrollment.

### Study design

This was a single-arm, phase I, open-label study conducted at two participating hospitals in China (ClinicalTrials.gov identifier: NCT04258527), which aimed to evaluate PK/PD characteristics, and preliminary safety and antitumor activity of pemigatinib in Chinese patients with FGF/FGFR1-3 altered, advanced solid tumors.

### Participants

Patients were ≥ 18 years old with unresectable advanced, recurrent or metastatic solid tumor bearing documented FGF/FGFR alterations, and had failed to prior therapy or no standard therapy available. Patients had at least one measurable lesion, an Eastern Cooperative Oncology Group (ECOG) performance status of 0 or 1, and an anticipated life expectancy of ≥ 12 weeks. Patients who previously received the selective FGFR inhibitor were excluded. Other key exclusion criteria included history of calcium and phosphate hemostasis disorder, and ophthalmologically confirmed corneal/retinal disease (with clinical significance).

### Study treatment

Patients received oral pemigatinib at 13.5 mg once daily every 3 weeks on an intermittent 2-weeks-on/1-week-off dosing schedule. Tumor response was assessed by investigator according to Response Evaluation Criteria in Solid Tumors (RECIST) v1.1 every 9 weeks after first dosing. If the patients were considered benefiting from the treatment by the investigator, pemigatinib could be continued until disease progression, unacceptable toxicity, initiation of other anti-tumor therapy, or for a maximum of 2-year treatment.

### Outcomes and assessments

The primary endpoint was PK/PD characteristics of pemigatinib. For assessment of PK characteristics, plasma samples were collected from all the patients at before each pemigatinib administration on days 1, 2, 8, 14, 15, and 16 during cycle 1, and immediately after, and 0.5 h ± 2 min, 1 h ± 15 min, 2 h ± 15 min, 4 h ± 15 min, 6 h ± 30 min, and 8 h ± 30 min after each pemigatinib administration on days 1 and 14 during cycle 1. Plasma pemigatinib concentrations were measured using the validated liquid chromatography–tandem mass spectrometry (LC–MS/MS) method. LC–MS/MS analysis for pemigatinib was carried out with a Sciex API 4000 mass spectrometer, coupled with an HPLC pump and an autosampler. PK parameters for evaluation included maximum serum concentration (C_max_), time to C_max_ (t_max_), clearance, volume of distribution (V_d_), elimination half-life (t_1/2_), etc.

Serum phosphate level was defined as the PD marker in the study for assessing FGFR inhibition. Serial blood samples were collected at before each pemigatinib administration on days 1, 2, 8, and 15 during cycle 1. Serum phosphate levels were measured using phosphomolybdate UV method (measuring range: 0.31‑20.0 mg/dL) by the central laboratory (Q2 Solutions [Beijing] Co., Ltd.).

The secondary endpoints were safety and efficacy including objective response rate (ORR), disease control rate (DCR), progression-free survival (PFS; duration between the first dosing of pemigatinib and the first documented progressive disease or death). Tumor was assessed per RECIST v1.1 by investigator at baseline, and every 9 weeks after first dosing. Safety was monitored up to 30 days after discontinuation of pemigatinib treatment. Adverse events (AEs) were graded per CTCAE v5.0. Treatment-emergent AEs (TEAEs; defined as an adverse event that emerged or worsened from first dosing to 30 days after pemigatinib treatment discontinuation) are reported. Sponsor-defined clinically notable TEAEs included hyperphosphatemia, hypophosphatemia, nail toxicities, and serous retinal detachment.

### Statistical analysis

All statistical analyses were conducted using SAS version 9.4 (or higher). The safety and efficacy were assessed in those patients who received at least 1 dose of pemigatinib. The PK characteristics were established from the PK analysis set (patients who had received at least one dose of pemigatinib and provided at least one post-baseline PK samples as per protocol).

Standard non-compartmental pharmacokinetic methods with PKanalix2020R1 (Lixoft, Antony, France) were applied to estimate PK parameters, including AUC, C_max_, T_max_, clearance (CL), V_d_, and t_1/2_. The apparent oral clearance at steady state (CL_ss_/F) was computed as Dose/AUC_ss,0-τ_, where F is the (unobserved) absolute bioavailability. It was included in the above relationship to indicate that the reported value was an apparent value based on extravascular administration. The accumulation ratio was computed as the ratio of AUC_ss,0–24_ (steady-state area under the curve from hour 0 to 24) to AUC_C1D1,0–24_ (area under the curve from hour 0 to 24 on Cycle 1, Day 1).

The Clopper-Pearson method was used to calculate the 95% confidence interval (CI) of ORR and DCR. The Kaplan–Meier method and the Brookmeyer and Crowley method were used to estimate PFS.

## Results

### Baseline characteristics

From February 20, 2020 to July 2, 2020, 25 patients were screened; a total of 12 patients were enrolled and received treatment (median age of 61 years [range: 27–69], 7 [58.3%] males, 6 [50.0%] ECOG PS 1, 6 [50.0%] metastatic solid tumors), including 4 (33.3%) patients with CRC, 3 (25%) gastric/gastroesophageal junction carcinoma, 2 (16.7%) esophageal carcinoma, 2 (16.7%) CCA, and 1 (8.3%) breast cancer. Five (5/12, 41.7%) patients received ≥ 3 lines of prior systemic anti-cancer treatment. All patients harbored one or more types of FGF/FGFR1-3 alterations including FGFR1 amplification (4/12, 33.3%), FGFR2 point mutation (3/12, 25.0%), FGFR1 point mutation (2/12, 16.7%), FGFR2 amplification (2/12, 16.7%), FGFR2 fusion (2/12, 16.7%), FGF3 point mutation (1/12, 8.3%), FGF3 amplification (1/12, 8.3%), FGF4 amplification (1/12, 8.3%), and FGF19 amplification (1/12, 8.3%). The demographics and baseline characteristics were listed in Table 1.

**Table 1:** Patients’ demographic and baseline characteristics

Characteristics	All patients (N = 12) n (%)
Age, years	
Median	61
Range	27–69
Sex	
Male	7 (58.3)
Female	5 (41.7)
ECOG performance status	
0	6 (50.0)
1	6 (50.0)
Prior treatment lines	
1	1 (8.3)
2	6 (50.0)
≥ 3	5 (41.7)
Cancer type	
Colorectal cancer	4 (33.3)
Gastric and gastroesophageal junction carcinoma	3 (25.0)
Cholangiocarcinoma	2 (16.7)
Esophageal carcinoma	2 (16.7)
Breast cancer	1 (8.3)
FGF/FGFR alteration	
FGFR1 amplification	4 (33.3)
FGFR2 amplification	2 (16.7)
FGF3 amplification	1 (8.3)
FGF4 amplification	1 (8.3)
FGF19 amplification	1 (8.3)
FGFR1 mutation	2 (16.7)
FGFR1 p.A354V	1 (8.3)
FGFR1 p.H466D	1 (8.3)
FGFR2 mutation	3 (25.0)
FGFR2 p.F276C	1 (8.3)
FGFR2 p.V691Cfs*2	1 (8.3)
NA	1 (8.3)
FGF3 mutations	1 (8.3)
NA	1 (8.3)
FGFR2 fusion	2 (16.7)
FGFR2-CCDC6 fusion	1 (8.3)
NA	1 (8.3)

Percentages might not sum to 100 because of rounding

*ECOG* Eastern Cooperative Oncology Group, *FGF* fibroblast growth factor, *FGFR* fibroblast growth factor receptor, *NA* not available

As of the data cutoff date of March 8, 2021, all patients discontinued treatment due to disease progression. The median duration of treatment was 62 days (range: 35–246) with median treatment cycle of 3.0 (range: 2–12). All patients were in compliance with pemigatinib dosing regimen.

### PK/PD characteristics

**Fig. 1:**
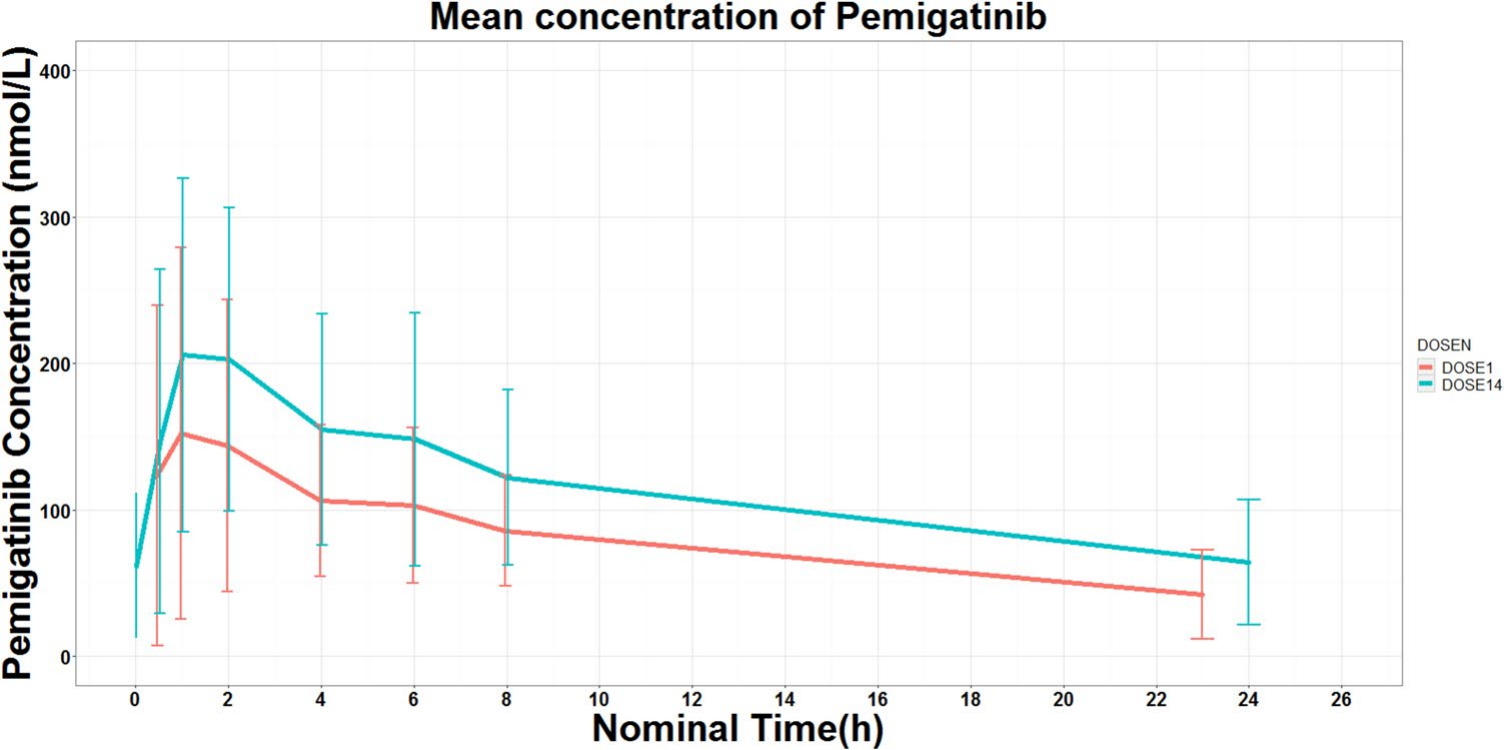
Mean plasma pemigatinib concentration–time curve (Mean ± SE) after single-dose or multiple-dose administration in Chinese patients with advanced cancer. DOSE 1: Mean Plasma pemigatinib concentration–time curve (Mean ± SE) after first-dose at Cycle 1 Day 1; DOSE 14: Mean Plasma pemigatinib concentration–time curve (Mean ± SE) at Cycle 1 Day 14 (steady state)

Twelve participants who received pemigatinib 13.5 mg daily on the intermittent schedule were assessed for PK on Cycle 1, Day 1 (C1D1) and Cycle 1, Day 14 (C1D14), respectively. Figure 1 presents pemigatinib concentration over time after dosing of pemigatinib during C1D1 and C1D14 (steady state). A summary of the PK parameters of pemigatinib for C1D1 and C1D14 (steady state) are presented in Table 2.

**Table 2:** Summary of the pharmacokinetic parameters for pemigatinib in Chinese patients with advanced solid tumor

**The C1D1 pharmacokinetic parameters**								
Dose (mg)	N		C_max_ (nmol/L)		t_max_ (h)		AUC_0-24_ (nmol/L)	
13.5	12		190.2±108.3 (153.9, 56.9)		1.52 (0.5, 8)		2242.8±861.1 (2133.8, 38.4)	
**The C1D14 (steady state) pharmacokinetic parameters**								
Dose (mg)	N	Cmax,ss(nmol/L)	tmax (h)	Cmin,ss(nmol/L)	CLss/F(L/h)	Vz/F (L)	AUCss,0-24(h·nmol/L)	t½ (h)
13.5	12	246.3±114.7(215.1, 46.6)	1.99 (0.5, 23.2)	59.9± 41.7(49.8, 69.7)	13.3 ±7.2(11.8, 54.2)	179±60.4(170.5, 33.7)	2935.1±1437.6 (2636.9, 49)	11.8 ±4.0(11.3, 34.3)

Values are presented in "mean ± STD (geometric mean, CV%)" except "median (min, max)" for t_max_

*C1D1* cycle 1 day 1, *C1D14* Cycle 1 day 14, *N* number of participants, *C*_*max*_ maximal serum concentration, *t*_*max*_ time to reach C_max_, *AUC*_*0-24*_ area under the curve from hour 0 to 24, *C*_*max,ss*_ maximum observed plasma concentration at steady state, *C*_*min*_ minimum observed concentration between dose time and dose time + Tau, *CL*_*SS*_/*F* Clearance adjusted by bioavailablity at steady state, *Vz/F* apparent oral volume of distribution, *AUC*_*ss,0–24*_ area under the curve from 0 to 24 h at steady state, *t*_*1/2*_ elimination half-life, *CV%* percent coefficient of variation

Following daily administration of 13.5 mg pemigatinib, it attained peak plasma concentrations after a median t_max_ of approximately 1.52 h at C1D1 and 1.99 h at steady state. The geometric mean terminal elimination half-life was 11.3 h. The steady-state geometric mean C_max_ and AUC were 215.1 nmol/L7 and 2636.9 h·nmol/L, respectively. The degree of drug accumulation was measured by the accumulation ratio, and its geometric mean value was 1.32.

PD characteristics were analyzed in 11 out of 12 patients (with 1 subject excluded due to lack of baseline serum phosphate data). The serum phosphate concentrations increased on days 8 and 15 of cycle 1 (mean: 2.25 mg/dL, CV% [percent coefficient of variation]: 31.3%) and decreased to baseline post 1 week intermission. During subsequent treatment, the serum phosphorus remained below or near the baseline levels.

### Efficacy

**Fig. 2:**
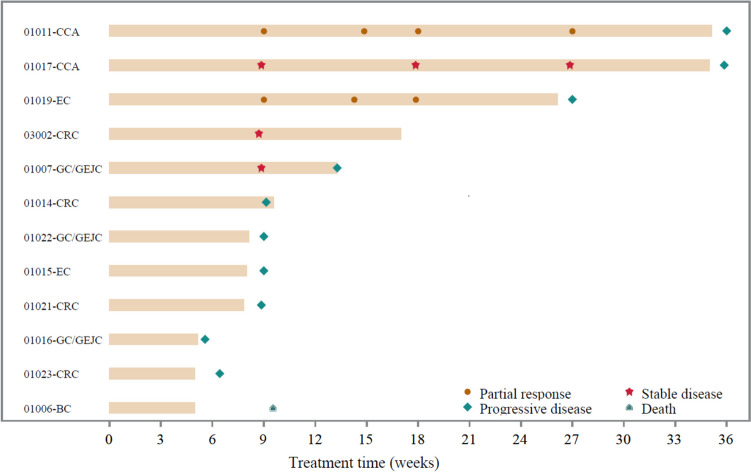
Tumor swimmer plot for all patients. Clinical response was evaluated using Response Evaluation Criteria in Solid Tumors v1.1 by investigators. Abbreviations: CRC, colorectal cancer; GC/GEJC, gastric or gastro-esophageal junction carcinoma; CCA, cholangiocarcinoma; EC, esophageal carcinoma; BC, breast cancer

Overall, confirmed partial response (PR) was observed in two patients per investigator assessments, including one patient with FGFR1-mutant (p.A354V) esophageal carcinoma and one with FGFR2-mutant (p.F276C) CCA (Fig. 2), which contributed to an ORR of 16.7% (95% confidence interval [CI], 2.1%–48.4%; Supplement Table S1). Additionally, three (3/12, 25.0%) patients achieved stable disease (SD), thus contributing to a DCR of 41.7% (95% CI, 15.2%–72.3%). As of the data cutoff, ten patients had progressed disease, one patient died due to disease progression 32 days after last dosing, and one patient was lost to follow-up. Median PFS was 2.1 months (95% CI, 1.5–6.2), with a median follow-up of 5.1 months (range, 1.5–9.3).

### Safety

All patients experienced TEAEs (Table 3), most frequently hyperphosphataemia (12/12, 100%), hyponatraemia (8/12, 66.7%), decreased appetite (6/12, 50.0%), hypoalbuminaemia (6/12, 50.0%), and anaemia (6/12, 50.0%). Three (3/12, 25.0%) patients had grade ≥ 3 TEAEs, including hyponatraemia, hypercalcaemia, gamma-glutamyltransferase increased, lipase increased, proteinuria, and arthralgia, each in one patient (1/12, 8.3%); two (2/12, 16.7%) patients experienced grade ≥ 3 treatment-related AEs. No serious TEAEs occurred. No TEAEs led to treatment interruption/discontinuation or death; one (1/12, 8.3%) patient had dose reduction due to proteinuria.

**Table 3:** Most common treatment-emergent adverse events (occurred in ≥ 25% of all patients)

Preferred terms	All patients (N = 12)	
	**Any grade** **n (%)**	**Grade ≥ 3** **n (%)**
Any treatment-emergent adverse event	12 (100)	3 (25.0)
Hyperphosphataemia	12 (100)	0
Hyponatraemia	8 (66.7)	1 (8.3)
Hypoalbuminaemia	6 (50.0)	
Decreased appetite	6 (50.0)	
Anaemia	6 (50.0)	
Low density lipoprotein increased	5 (41.7)	0
Blood alkaline phosphatase increased	5 (41.7)	0
Hypochloraemia	4 (33.3)	0
Gamma-glutamyltransferase increased	4 (33.3)	1 (8.3)
Weight decreased	4 (33.3)	0
Blood lactate dehydrogenase increased	4 (33.3)	0
Blood bilirubin increased	4 (33.3)	0
Diarrhoea	4 (33.3)	0
Proteinuria	4 (33.3)	1 (8.3)
Hypophosphataemia	3 (25.0)	0
Hypercalcaemia	3 (25.0)	1 (8.3)
Aspartate aminotransferase increased	3 (25.0)	0
White blood cell count decreased	3 (25.0)	0
Vitamin D decreased	3 (25.0)	0
Blood parathyroid hormone decreased	3 (25.0)	0
Blood creatinine increased	3 (25.0)	0
Nausea	3 (25.0)	0
Abdominal pain	3 (25.0)	0
Asthenia	3 (25.0)	0
Alopecia	3 (25.0)	0

Adverse events were classified according to *Medical Dictionary for Regulatory Activities* and graded according to the National Cancer Institute Common Terminology Criteria for Adverse Events, version 5.0. Treatment-emergent adverse events occurring in 25% or more of patients are shown in a descending order

All patients experienced at least one sponsor-defined clinically-notable TEAE, all being grade 1 or 2 in severity, including hyperphosphatemia (12/12, 100%), hypophosphatemia (3/12, 25.0%), nail toxicity (2/12, 16.7%), nail disorder (2/12, 16.7%) and nail discolouration (1/12, 8.3%).

## Discussion

The safety and efficacy with pemigatinib has been previously reported in Chinese patients with CCA [19]. This phase I trial is the first to disclose the PK/PD, preliminary safety and efficacy with pemigatinib in Chinese patients with diverse advanced solid tumors. The results suggested that PK/PD characteristics and safety with pemigatinib in Chinese patients were generally consistent with that in Western population. Favorable clinical benefit was observed across several FGF/FGFR1-3 altered, advanced, solid tumors.

The PK data indicated rapid absorption of pemigatinib in Chinese patients and consistent PK parameters between single and multiple dosing. Pemigatinib exposures at steady state in this study were generally consistent with previous data in Western population (INCB 54828–101) [18], with C_max,ss_ (geomean [CV%]) of 215.1 nM [64.8%] vs 236 nM [56.4%] and AUC_ss,0–24_ (geomean [CV%]) of 2636.9 h·nM [53.8%] vs 2620 h·nM [54.1%]. Serum phosphate was assessed as the PD marker and its concentration increased at first cycle and decreased to and remained around baseline levels after 1-week intermission. The serum phosphate concentration change from baseline (geomean [CV%]) was 2.15 (32.9%) in this study and was 2.6 (40.4%) in Western population (INCB 54828–101) [18]. Those findings indicated PK/PD characteristics were generally similar between Chinese and Western populations (Supplement Table S2).

Preliminary anti-tumor activity was observed with pemigatinib in this previously treated patient population: 3 out of 12 patients achieved SD and 2 of 12 patients achieved PR. The overall efficacy of pemigatinib in the Chinese patients was similar to Western patients as reported in the phase I/II Fight-101 study (Supplement Table S2) [18] and slightly higher than the Japanese population as reported in the phase I Fight-102 study [20] (ORR: 16.7% vs 9.4% vs 4.0%; DCR: 41.7% vs 40.6% vs 40.0%), which might be attributed to the small sample size in the present study. Here it should be also noted that, compared to the present study, there were more patients with relatively poor ECOG PS (ECOG PS 1–2: 50% vs 80.5%) and more previously heavily pretreated patients (patients with ≥ 3 prior lines of therapy: 41.7% vs 76.6%) in Fight-101 study [18].

Accumulating data suggest that FGF/FGFR alterations are associated with tumor sensitivity to FGFR inhibitors although their underlying role has not been fully elucidated [21–23]. It should be noted that in the present study the two patients with overall response of PR were with FGFR1 and FGFR2 point mutations, and thus the ORR for the patients with FGFR point mutations was 33.3% (2/6). The previous efficacy data from Fight-101 study with pemigatinib in Western patients showed that ORR for patients with FGFR mutations was 23.1% (3/13), secondary to the patients with FGFR fusions/rearrangements (25.0%, 5/20) [18]. In phase I Fight-102 study in the Japanese population, objective clinical response was reported only in one (1/25, 4.0%) patient bearing FGFR2 amplification out of the 25 patients assessed [20]. Additionally, the two point mutations detected in the patients with PR, FGFR1 p.A354V and FGFR2 p.F276C, lie in the receptors’ extra-cellular domain and have not been previously reported. The present results indicate that pemigatinib may have anti-cancer activity in other solid tumors bearing certain FGFR point mutations other than FGFR2 fusion/rearrangement-positive CCA as approved by the regulatory in multiple regions. However, more work still needs to be done to clarify the underlying mechanism of those two specific point mutations in response to pemigatinib.

The safety results showed that the investigated pemigatinib regimen was generally well tolerated in Chinese patients with FGF/FGFR-altered solid tumors. Most TEAEs were grade 1–2 in severity and were manageable with supportive care; only 1 out of 12 patient needed dose reduction. The safety profile was generally consistent with previous data from Western patients and Japanese patients treated with pemigatinib [15, 18, 20]; no new safety signals were observed even when compared with other selective FGFR inhibitors [24–28]. The incidence of any-grade hyperphosphataemia, which was the most common AE of selective FGFR inhibitors due to its mechanism of action, was numerically higher when compared with the safety data in Western patients and Japanese patients (incidence of any grade: 100% vs 75% vs 76%, incidence of grade ≥ 3: 0 vs 2.3% vs not available) [18, 20], but all were grade 1–2 in severity and occurred only in treatment cycle 1 in this study. With respect to other frequently-observed AEs with selective FGFR inhibitors including skin and mucosal dryness, nail and ocular toxicity [18, 25–28], all such AEs were with low incidence and were grade 1–2 in this study. Of note, only one patient experienced ocular toxicity, vision blurred of grade 1, which was different from the ocular toxicity profile of Western patients with solid tumors in FIGHT-101 study [18] including dry eye, eyelash changes, vision blurred. Those results indicated that pemigatinib possibly have a better ocular safety profile in Chinese patients than that in Western patients, which however couldn’t be determined as limited by the small sample size.

Limitations exist in this study. Firstly, as the study was designed as an open-label phase I study, the data had inherent limitations. In addition, the sample size was small with only 12 patients enrolled into the study. Speaking of different tumor types, the number of patients enrolled is even more limited.

In conclusion, in this phase I study, pemigatinib had similar PK/PD characteristics to western population and an acceptable safety profile in Chinese patients, with no new safety signals identified. Preliminary promising antitumor activity was observed in Chinese patients with FGF/FGFR1-3 altered, advanced, solid tumors.

### Supplementary Information

Below is the link to the electronic supplementary material.

Supplementary file1 (DOCX 17 KB)

Supplementary file2 (DOCX 18 KB)

## Data Availability

The anonymized data that support the findings of this study are available from the corresponding author upon reasonable request.

## References

[1] Krook MA, Reeser JW, Ernst G, Barker H, Wilberding M, Li G (2021). Fibroblast growth factor receptors in cancer: genetic alterations, diagnostics, therapeutic targets and mechanisms of resistance. Br J Cancer.

[2] Lengyel CG, Hussain S, Seeber A, Jamil Nidhamalddin S, Trapani D, Habeeb BS (2022). FGFR Pathway Inhibition in Gastric Cancer: The Golden Era of an Old Target?. Life.

[3] Zhou Z, Liu Z, Ou Q, Wu X, Wang X, Shao Y (2021). Targeting FGFR in non-small cell lung cancer: implications from the landscape of clinically actionable aberrations of FGFR kinases. Cancer Biol Med.

[4] Garje R, An J, Obeidat M, Kumar K, Yasin HA, Zakharia Y (2020). Fibroblast Growth Factor Receptor (FGFR) Inhibitors in Urothelial Cancer. Oncologist.

[5] Fromme JE, Schmitz K, Wachter A, Grzelinski M, Zielinski D, Koppel C (2018). FGFR3 mRNA overexpression defines a subset of oligometastatic colorectal cancers with worse prognosis. Oncotarget.

[6] Kiladjian J-J, Shitara K, Rosen LS, Rha SY, He A, Oh D-Y (2021). A Phase 2 Study of Futibatinib (TAS-120) in Patients with Myeloid or Lymphoid Neoplasms Harboring Fibroblast Growth Factor Receptor (FGFR) 1 Rearrangements. Blood.

[7] Mazzaferro V, El-Rayes BF, Droz Dit Busset M, Cotsoglou C, Harris WP, Damjanov N (2019). Derazantinib (ARQ 087) in advanced or inoperable FGFR2 gene fusion-positive intrahepatic cholangiocarcinoma. Br J Cancer.

[8] Dehghanian F, Alavi S (2021). Molecular mechanisms of the anti-cancer drug, LY2874455, in overcoming the FGFR4 mutation-based resistance. Sci Rep.

[9] Franovic A, Mohan A, Uryu S, Wu Q, Jiang P, Miller N et al (2022) Activity of KIN-3248, a next-generation pan-FGFR inhibitor, against acquired FGFR-gatekeeper and molecular-brake drug resistance mutations. J Clin Oncol 40(4_suppl):461–461

[10] Chae YK, Hong F, Vaklavas C, Cheng HH, Hammerman P, Mitchell EP (2020). Phase II Study of AZD4547 in Patients With Tumors Harboring Aberrations in the FGFR Pathway: Results From the NCI-MATCH Trial (EAY131) Subprotocol W. J Clin Oncol.

[11] Cleary JM, Iyer G, Oh D-Y, Mellinghoff IK, Goyal L, Ng MCH et al (2020) Final results from the phase I study expansion cohort of the selective FGFR inhibitor Debio 1,347 in patients with solid tumors harboring an FGFR gene fusion. J Clin Oncol 38(15_suppl):3603–3603

[12] Loriot Y, Necchi A, Park SH, Garcia-Donas J, Huddart R, Burgess E (2019). Erdafitinib in Locally Advanced or Metastatic Urothelial Carcinoma. New Eng J Med.

[13] Javle M, Lowery M, Shroff RT, Weiss KH, Springfeld C, Borad MJ (2018). Phase II Study of BGJ398 in Patients With FGFR-Altered Advanced Cholangiocarcinoma. J Clin Oncol.

[14] Javle MM, Roychowdhury S, Kelley RK, Sadeghi S, Macarulla T, Waldschmidt DT et al (2021) Final results from a phase II study of infigratinib (BGJ398), an FGFR-selective tyrosine kinase inhibitor, in patients with previously treated advanced cholangiocarcinoma harboring an FGFR2 gene fusion or rearrangement. J Clin Oncol 39(3_suppl):265–265

[15] Abou-Alfa GK, Sahai V, Hollebecque A, Vaccaro G, Melisi D, Al-Rajabi R (2020). Pemigatinib for previously treated, locally advanced or metastatic cholangiocarcinoma: a multicentre, open-label, phase 2 study. Lancet Oncol.

[16] Liu PCC, Koblish H, Wu L, Bowman K, Diamond S, DiMatteo D (2020). INCB054828 (pemigatinib), a potent and selective inhibitor of fibroblast growth factor receptors 1, 2, and 3, displays activity against genetically defined tumor models. PLoS ONE.

[17] Gotlib J, Kiladjian J-J, Vannucchi A, Rambaldi A, Reiter A, Shomali W (2021). A Phase 2 Study of Pemigatinib (FIGHT-203; INCB054828) in Patients with Myeloid/Lymphoid Neoplasms (MLNs) with Fibroblast Growth Factor Receptor 1 (FGFR1) Rearrangement (MLN FGFR1). Blood.

[18] Subbiah V, Iannotti NO, Gutierrez M, Smith DC, Féliz L, Lihou CF (2022). FIGHT-101, a first-in-human study of potent and selective FGFR 1–3 inhibitor pemigatinib in pan-cancer patients with FGF/FGFR alterations and advanced malignancies. Ann Oncol.

[19] Shi GM, Huang XY, Wen TF, Song TQ, Kuang M, Mou HB (2022). Pemigatinib in previously treated Chinese patients with locally advanced or metastatic cholangiocarcinoma carrying FGFR2 fusions or rearrangements: A phase II study. Cancer Med.

[20] Kuboki Y, Furukawa M, Takahashi Y, Mizuno N, Hara H, Ueno M et al (2019) Preliminary results from fight-102: a phase 1 study of pemigatinib in Japanese patients with advanced malignancies. Ann Oncol 30:vi125 10.1002/cam4.5798 PMC10225205 37000035

[21] Babina IS, Turner NC (2017). Advances and challenges in targeting FGFR signalling in cancer. Nat Rev Cancer.

[22] Karkera JD, Cardona GM, Bell K, Gaffney D, Portale JC, Santiago-Walker A (2017). Oncogenic Characterization and Pharmacologic Sensitivity of Activating Fibroblast Growth Factor Receptor (FGFR) Genetic Alterations to the Selective FGFR Inhibitor Erdafitinib. Mol Cancer Ther.

[23] Zheng J, Zhang W, Li L, He Y, Wei Y, Dang Y (2022). Signaling Pathway and Small-Molecule Drug Discovery of FGFR: A Comprehensive Review. Front Chem.

[24] Bahleda R, Italiano A, Hierro C, Mita A, Cervantes A, Chan N (2019). Multicenter Phase I Study of Erdafitinib (JNJ-42756493), Oral Pan-Fibroblast Growth Factor Receptor Inhibitor, in Patients with Advanced or Refractory Solid Tumors. Clin Cancer Res.

[25] Bahleda R, Meric-Bernstam F, Goyal L, Tran B, He Y, Yamamiya I (2020). Phase I, first-in-human study of futibatinib, a highly selective, irreversible FGFR1-4 inhibitor in patients with advanced solid tumors. Ann Oncol.

[26] Meric-Bernstam F, Bahleda R, Hierro C, Sanson M, Bridgewater J, Arkenau HT (2022). Futibatinib, an Irreversible FGFR1-4 Inhibitor, in Patients with Advanced Solid Tumors Harboring FGF/FGFR Aberrations: A Phase I Dose-Expansion Study. Cancer Discov.

[27] Nogova L, Sequist LV, Perez Garcia JM, Andre F, Delord JP, Hidalgo M (2017). Evaluation of BGJ398, a Fibroblast Growth Factor Receptor 1–3 Kinase Inhibitor, in Patients With Advanced Solid Tumors Harboring Genetic Alterations in Fibroblast Growth Factor Receptors: Results of a Global Phase I, Dose-Escalation and Dose-Expansion Study. J Clin Oncol.

[28] Saka H, Kitagawa C, Kogure Y, Takahashi Y, Fujikawa K, Sagawa T (2017). Safety, tolerability and pharmacokinetics of the fibroblast growth factor receptor inhibitor AZD4547 in Japanese patients with advanced solid tumours: a Phase I study. Invest New Drugs.

