# Supplementary material for: Pharmacokinetics, pharmacodynamics and efficacy of pemigatinib (a selective inhibitor of fibroblast growth factor receptor 1–3) monotherapy in Chinese patients with advanced solid tumors: a phase i clinical trial
Source: Invest New Drugs. 2023 Oct 27;41(6):808–15. doi: 10.1007/s10637-023-01396-x (PMC10663244; doi:10.1007/s10637-023-01396-x)
Supplement: Supplementary file 1 — Supplementary file1 (DOCX 17 KB) [file 10637_2023_1396_MOESM1_ESM.docx]

**SUPPLEMENTARY METHODS**

1. **Pharmacokinetics measurement**

Plasma pemigatinib concentrations were measured using the liquid chromatography-tandem mass spectrometry method. Pharmacokinetics method was developed and validated for the detection and quantitation of pemigatinib in human serum, LC-MS/MS analysis for pemigatinib was carried out with a Sciex API 4000 mass spectrometer, coupled with an HPLC pump and an autosampler. The chromatographic separation was achieved for pemigatinib on an ACE 5 C18-PFP, 50 x 2.1 mm, 5 μm particle size, with gradient elution. The mass spectrometer was operated in positive ESI mode, and the resolution setting used was unit for both Q1 and Q3. The multiple reaction monitoring (MRM) transition was m/z 488.1—401.2 for pemigatinib, and the MRM transition was m/z 501.5—414.3 for the internal standard, INCB055074. Peak-area integrations were performed using Analyst software (version 1.7.0) from Applied Biosystems, and regressions were carried out in Watson Bioanalytical LIMS (version 7.5) from Thermo Scientific. Concentrations were calculated using 10 concentration level curves ranging from 1 nM to 1000 nM with weighted linear regression for pemigatinib, according to the following formula: y = mx + b (weighting factor = 1/x2), where x = pemigatinib concentration in nM, y = Peak-area ratio, m = Slope, and b = Intercept. The lower limit of quantitation was 1 nM, and the calibration curve ranged from 1 to 1000 nM for pemigatinib.

1. **Pharmacodynamics measurement**

Serum samples were analyzed for the concentration of serum phosphate using phosphomolybdate UV method by the central laboratory (Q2 Solutions (Beijing) Co., Ltd.). The concentration of phosphomolybdate formed is directly proportional to the inorganic phosphate concentration and is measured photometrically. Phosphorus quantification was determined in human plasma on Roche/Hitachi cobas c systems. Measuring range was 0.10‑6.46 mmol/L (0.31‑20.0 mg/dL). Reagents: Phosphate (Inorganic) ver.2 (250 tests) Catalouge:03183793 122.
