# Supplementary material for: Pharmacokinetics, pharmacodynamics and efficacy of pemigatinib (a selective inhibitor of fibroblast growth factor receptor 1–3) monotherapy in Chinese patients with advanced solid tumors: a phase i clinical trial
Source: Invest New Drugs. 2023 Oct 27;41(6):808–15. doi: 10.1007/s10637-023-01396-x (PMC10663244; doi:10.1007/s10637-023-01396-x)
Supplement: Supplementary file 2 — Supplementary file2 (DOCX 18 KB) [file 10637_2023_1396_MOESM2_ESM.docx]

**Supplementary tables**

Table S1. Antitumor activity per RECIST v1.1 as assessed by investigator

|  | **Number of patients (%)** |
| --- | --- |
| **Best overall confirmed response, n (%)** | |
| Complete response | 0 |
| Partial response | 2 (16.7%) |
| Stable disease | 3 (25.0%) |
| Progressive disease | 6 (50.0%) |
| Not assessed^a^ | 1 (8.3%) |
| **Objective response rate** |  |
| n (%) | 2 (16.7%) |
| 95% CI | (2.1% - 48.4%) |
| **Disease control rate** |  |
| n (%) | 5 (41.7%) |
| 95% CI | (15.2% - 72.3%) |

^a^Tumor assessment was not available in one patient.

Table S2. Pharmacokinetics/pharmacodynamics characteristics and antitumor activity with pemigatinib in Chinese patients versus Western patients.

|  | **Chinese patients^a^** | **Western patients^b^** |
| --- | --- | --- |
| **Pharmacokinetics** | | |
| C_max,ss_, geomean (CV%) | 215.1 nM (64.8%) | 236 nM (56.4%) |
| AUC_ss,0-24_, geomean (CV%) | 2636.9 h·nM (53.8%) | 2620 h·nM (54.1%) |
| **Pharmacodynamics** | | |
| Serum phosphate concentration change (mg/dL)^c^,  geomean (CV%) | 2.15 (32.9%) | 2.6 (40.4%) |
| **Antitumor activity** | | |
| Objective response rate, % | 16.7% | 9.4% |
| Disease control rate, % | 41.7% | 40.6% |

Abbreviations: C_max,ss_, maximum observed plasma concentration at steady state; AUC_ss,0-24_, area under the curve from 0 to 24 hour at steady state; CV%, percent coefficient of variation.

^a^The data were derived from the present study.

^b^Subbiah, V., et al. (2022). FIGHT-101, a first-in-human study of potent and selective FGFR 1-3 inhibitor pemigatinib in pan-cancer patients with FGF/FGFR alterations and advanced malignancies. Ann Oncol 33 (5), 522-533.

^c^The change of serum phosphate concentrations on days 8 and 15 of cycle 1 from baseline.
